# Factors associating with disability of non-specific low back pain in different subgroups: A hierarchical linear regression analysis

**DOI:** 10.1038/s41598-021-97569-w

**Published:** 2021-09-14

**Authors:** Takahiro Miki, Daisuke Higuchi, Tsuneo Takebayashi, Mina Samukawa

**Affiliations:** 1 Department of Rehabilitation, Sapporo Maruyama Orthopedic Hospital, N7, W27, Chuo, Sapporo, Hokkaido 006-0007 Japan; 2 Faculty of Health Sciences, Hokkaido University, Sapporo, Japan; 3 Department of Physical Therapy, Faculty of Health Care, Takasaki University of Health and Welfare, Takasaki, Japan; 4 Department of Orthopedic, Sapporo Maruyama Orthopedic Hospital, Sapporo, Japan

**Keywords:** Health care, Human behaviour

## Abstract

This study aimed to explore factors associating with disability, which means physical impairment affecting a person’s mobility, capacity, stamina, or agility, of non-specific low back pain (NSLBP) of the acute and non-acute groups. Two hundred thirty-five patients with NSLBP of less than 8 weeks’ duration as acute groups (n = 124) and more than 8 weeks’ duration as non-acute group (n = 111) were recruited. It was collected data on pain intensity, disability and psychosocial factors, including pain catastrophising, fear of movement and pain self-efficacy. Disability was measured Roland Morris Disability Questionnaire. A hierarchical multiple regression analysis was performed to analyse factors associating with disability of the acute and non-acute groups. The Result was that explanatory power increased with each additional variable of the order of demographic characteristics, pain intensity and psychosocial factors for both groups. Pain intensity, pain catastrophising and pain self-efficacy had significant explanatory power, with pain self-efficacy having the most significant association on the acute group. Only pain self-efficacy having the most significant association on disability of the non-acute group. In conclusion, the factors associating with disability differed depending on the duration of the disease, and pain self-efficacy might be one of the factors associating with disability of patients with NSLBP.

## Introduction

Low back pain (LBP) has become a global problem, and medical costs are increasing every year^1^. 80–90% of LBP are classified as non-specific low back pain (NSLBP), which cannot be attributed to a structural specific problem^2^. In NSLBP, one of the most important outcomes is disability which means physical impairment affecting a person’s mobility, physical capacity, stamina, or agility, and it is widely known that many factors such as patient’s character, pain intensity and psychosocial factors influence disability of NSLBP^3^. In particular, fear of movement, pain catastrophic thinking and self-efficacy have been widely reported as psychosocial factors associating with NSLBP in the previous studies^4,5^. Alamam et al. reported that the factors that contributed to chronicity after 1 year were high fear of movement and older age and the intensity of pain at the beginning of the disease^6^. Fear of movement is an important predictor of failure to return to work and it is believed to contribute to disability of NSLBP^7,8^. In terms of self-efficacy, it is related to pain intensity and disability, but a correlation between low self-efficacy and physical function has been reported^9^. For example, lumbar potential and stability during lifting movements were reduced of NSLBP patients with low self-efficacy^10^. In addition, Wertli et al. reported that catastrophic thinking is associated with disability and may contribute to chronicity^11^.

Thus, from previous studies, it is clear that many factors are involved in disability of NSLBP. However, no study has examined which factors have more impact on disability of NSLBP. Furthermore, most of such studies were on non-acute NSLBP, not acute NSLBP. The important aspect of NSLBP is to take appropriate action of the acute phase to prevent it from becoming chronicity. This is because it is difficult to identify and remedy the causes of chronic conditions^12^. Depending on the duration of the disease, factors associating with disability of NSLBP may differ. Identifying factors that influence disability of the acute and non-acute phase provides appropriate interventions for at each phase and important information to prevent chronicity.

Therefore, this study explores which factors influence disability of acute and non-acute phases of NSLBP. We hypothesised that the factors associating with disability differed depending on the duration of the disease.

## Materials and methods

### Participants

Participants were collected from the two medical institutions from January 2019 to December 2020. Written informed consent was obtained from the patients before the study. The study was conducted in compliance with the Declaration of Helsinki and was conducted with the approval of the Ethics Committee of Sapporo Maruyama Orthopaedic Hospital (no. 0039) and the Faculty of Health Sciences, Hokkaido University (approved no. 20-58). The inclusion criteria of the participants were: (1) over 20 years old; (2) diagnosed as NSLBP by an orthopaedic surgeon, with pain occurring in the lumbosacral region with radiation limited above the knee, with or without referred pain and signs of nerve root compromise^2^; (3) no use of painkillers at the initial physiotherapy session and (4) being able to understand the Japanese language well enough to complete the questionnaires independently. The exclusion criteria included (1) presence of spinal deformities; (2) any history of surgery on the spine; (3) pregnancy; (4) rheumatological and inflammatory disease; and (5) other serious pathologies (e.g., pyogenic spondylitis and cauda equina syndrome). Participants were divided into two groups: the acute NSLBP group with an onset of fewer than 8 weeks (56 days) and the non-acute NSLBP group with more than 57 days^13^.

### Procedures

This cross-sectional study compared two subgroups of NSLBP at two hospitals was performed according to the STROBE (Strengthening the Reporting of Observational Studies in Epidemiology) statement. We collected data on age, sex, height, weight, medical diagnosis and duration of symptoms from medical records. Furthermore, we collected data on pain intensity, disability and psychosocial factors by patient-reported outcome measures (PROMs). For all participants, the following PROMs were evaluated immediately before the initial physiotherapy session: (1) an 11-point numerical rating scale (NRS) for pain intensity^14,15^; (2) Roland Morris Disability Questionnaire (RMDQ) for disability due to LBP^3^; (3) Pain Catastrophising Scale (PCS) for pain catastrophising^16^; (4) Tampa Scale for Kinesiophobia (TSK) for fear of movement^17^; and (5) Pain Self-Efficacy Questionnaire (PSEQ) for pain self-efficacy^18^.

### Measures

#### Pain intensity

NRS was used for pain intensity. The 11-point NRS was used to measure pain intensity^14,15^ to determine average pain intensity on the evaluation day. A score of 0 indicates no pain, and a score of 10 indicates the worst imaginable pain. Alghadir et al. assessed knee pain intensity with multiple scales, including the NRS, and demonstrated that NRS was valid^19^.

#### Disability

The Japanese version of the RMDQ, which contains 24 dichotomous scales (No = 0; Yes = 1), was used^20^. A higher total score indicates more significant functional impairment due to LBP. Concurrent validity of the Japanese RMDQ, high internal consistency (Cronbach's alpha for all items = 0.86) and high test–rest reliability (0.95) have been clarified^20^.

#### Pain catastrophising

To assess pain catastrophising, the Japanese version of the PCS, consisting of 13 items from 0 to 4, was used^21^. The total score is 52, with a higher total score indicating more significant deficits of pain catastrophising. 30 is the cut-off point for a clinically relevant level of catastrophising^16^. The construct validity and high internal consistency of the Japanese version of the PCS (Cronbach alpha for all items = 0. 89) were shown^21^.

#### Fear of movement

The Japanese version of the TSK, consisting of 17 items from 1 to 4 and a total score of 68, was used. A higher total score indicates strong fear of movement. 37 is the cut-off point for it^22^. The concurrent validity and high internal consistency of the Japanese version of the TSK (Cronbach alpha for all items = 0.85) have been confirmed^22^.

#### Pain self-efficacy

The Japanese version of PSEQ, consisting of 10 items from 0 to 6, was used for pain self-efficacy^23^. A high total score indicates stronger self-efficacy for pain. The concurrent validity and high internal consistency of the Japanese version of the PSEQ (Cronbach alpha for all items are more than 0.50) have been confirmed^23^. There is no cut-off value.

### Data analysis

We excluded subjects with missing data in the demographic characteristics and measures (n = 25). The differences were compared between the acute NSLBP and non-acute NSLBP groups using the Welch’s test. The association between disability and other factors was examined for each group using Pearson's product-moment correlation coefficient. For this study, r-values of ≤ 0.40, 0.40–0.75 and ≥ 0.75 were considered to indicate weak or no correlation, moderate correlation and strong correlation, respectively^24^. Furthermore, a hierarchical multiple regression analysis was performed to analyse factors associating with disability for the acute and non-acute groups, respectively.

The object (dependent) variable included the RMDQ for disability, and the explanatory (independent) variables included the characteristics of the participants, pain intensity and psychological factors (PCS, TSK and PESQ). Demographic characteristics including age, body weight and BMI were first entered (step 1) as control variables, followed by pain intensity in the second step (step 2). Finally, the psychological factors, including the scores of PCS, TSK and PESQ, were added in the third step (step 3) to test unique associations between the psychological factors and patients’ disability beyond the effects of patients’ demographic characteristics and pain intensity. HAD ver. 16.1 (Hiroshi Shimizu, Nishinomiya, Japan) was used to perform all statistical analyses^25^. All p-values are two-sided. The alpha level was set at 5%.

### Ethics statement

Written informed consent was obtained from the patients before the study. The study was conducted in compliance with the Declaration of Helsinki and was conducted with the approval of the Ethics Committee of Sapporo Maruyama Orthopaedic Hospital (no. 0039) and the Faculty of Health Sciences, Hokkaido University (approved no. 20-58).

## Results

### Demographic data

The total number of participants was 235 (124 participants of the acute NSLBP group and 111 participants of the NSLBP non-acute group) from two facilities. There was no significant difference in the participants' demographic characteristics between the two facilities. Table 1 shows the participants’ demographic characteristics for each group.

**Table 1:** Characteristics of the participants.

Patients	Acute NSLBP (n = 124)	Non-acute NSLBP (n = 111)
Age (year), mean (SD)	54.56 (15.20)	58.26 (15.81)
Gender (n of men), (%)	51 (41.1)	43 (38.7)
Height (cm), mean (SD)	162.28 (8.97)	159.32 (8.04)
Weight (kg), mean (SD)	63.15 (12.43)	60.89 (11.41)
BMI	23.87 (3.79)	23.92 (3.82)
Pain intensity over low back (0–10), mean (SD)	3.90 (2.30)	4.23 (2.08)
Roland-Morris Disability Questionnaire (0–24), mean (SD)	4.97 (4.15)	5.60 (4.45)
Pain Catastrophizing Scale (0–52), mean (SD)	20.92 (9.79)	22.18 (10.08)
Tampa Scale for Kinesiophobia (17–68), mean (SD)	35.83 (5.91)	36.01 (7.21)
Pain Self-Efficacy Questionnaire (0–60), mean (SD)	39.87 (11.65)	41.55 (10.90)

### Comparison of intergroup pain intensity, disability and psychosocial factors

Table 2 presents a comparison of pain intensity, disability and psychosocial factors between the acute and non-acute groups. There was no significant difference between the two groups.

**Table 2:** Comparison of intergroup pain intensity, disability and psychosocial factors.

Patients	Acute NSLBP (n = 124)	Non-acute NSLBP (n = 111)	P-value	95% Confidence interval
Pain intensity over the low back (0–10), mean (SD)	3.90 (2.31)	4.23 (2.08)	0.23	− 0.22 to 0.90
Roland-Morris Disability Questionnaire (0–24), mean (SD)	4.97 (4.15)	5.60 (4.45)	0.26	− 0.48 to 1.75
Pain Catastrophizing Scale (0–52), mean (SD)	20.93 (9.79)	22.18 (10.08)	0.33	− 1.30 to 3.81
Tampa Scale for Kinesiophobia (17–68), mean (SD)	35.83 (5.91)	36.02 (7.21)	0.82	− 1.52 to 1.90
Pain Self-efficacy Questionnaire (0–60), mean (SD)	39.87 (11.65)	41.55 (10.90)	0.25	− 1.22 to 4.58

### Correlation of disability, pain intensity and psychological factors

Table 3 shows a matrix of the correlations for the acute NSLBP and non-acute NSLBP groups. Positive indicates a positive correlation, and negative indicates a negative correlation. Moderately significant correlations were found between RDQ and PSEQ (r = − 0.409) and between TSK and PCS (r = 0.439) of the acute NSLBP group. Only RDQ and PSEQ (r = − 0.520) had a moderate or significant correlation of the non-acute NSLBP group.

**Table 3:** A matrix of the correlations.

	1	2	3	4	5	6	7
**Acute NSLBP group (n = 124)**							
1. Age	1.000						
2. BMI	− 0.041	1.000					
3. LBP	0.009	− 0.039	1.000				
4. PCS	− 0.073	− 0.045	0.062	1.000			
5. TSK	− 0.028	0.172	0.044	0.439**	1.000		
6. PSEQ	0.025	− 0.006	0.027	− 0.313**	− 0.342**	1.000	
7. RDQ	− 0.001	0.016	0.272**	0.317**	0.207**	− 0.409**	1.000
**Non-acute NSLBP group (n = 111)**							
1. Age	1.000						
2. BMI	0.133	1.000					
3. LBP	− 0.075	− 0.007	1.000				
4. PCS	− 0.032	0.144	0.363**	1.000			
5. TSK	0.172	0.043	0.100	0.314**	1.000		
6. PSEQ	− 0.203*	0.035	− 0.148	− 0.228*	− 0.228*	1.000	
7. RDQ	0.158	0.095	0.276**	0.376**	0.334**	− 0.520**	1.000

*p < 0.05, **p < 0.01.

*NSLBP* non-specific low back pain, *LBP* low back pain, *BMI* Body Mass Index, *PCS* Pain Catastrophizing Scale, *TSK* Tampa Scale for Kinesiophobia, *PESQ* Pain Self Efficacy Questionnaire, *RMDQ* Roland Morris Disability Questionnaire.

### Hierarchical multiple regression analysis

Tables 4 and 5 show the hierarchical multiple regression analysis results for the acute NSLBP and non-acute NSLBP groups.

**Table 4:** Results of hierarchical linear regression analysis for acute NSLBP group.

	Dependent variable: Disability		
	Step 1 (β)	Step 2 (β)	Step 3 (β)
Gender	0.000	− 0.050	− 0.409
Age	− 0.001	− 0.009	0.016
BMI	0.016	0.018	0.032
LBP		0.282**	0.280**
PCS			0.209**
TSK			− 0.035
PSEQ			− 0.361**
R^2^	0.00	0.077	0.284
ΔR^2^	0.00	0.077	0.207

*p < 0.05, **p < 0.01.

*β* Standardized coefficients, *NSLBP* non-specific low back pain, *LBP* low back pain, *BMI* Body Mass Index, *PCS* Pain Catastrophizing Scale, *TSK* Tampa Scale for Kinesiophobia, *PESQ* Pain Self Efficacy Questionnaire, *RMDQ* Roland Morris Disability Questionnaire, *R*^*2*^ the proportion of explained variance in dependent variable by the model, *ΔR*^*2*^ the change in R^2^ values from one model to another.

**Table 5:** Results of hierarchical linear regression analysis for non-acute NSLBP group (n = 111).

	Dependent variable: Disability		
	Step 1 (β)	Step 2 (β)	Step 3 (β)
Gender	0.080	0.072	0.066
Age	− 0.138	0.161	0.045
BMI	0.081	0.080	0.077
LBP		0.288**	0.139
PCS			0.173
TSK			− 0.156
PSEQ			− 0.416**
R2	0.13	0.119	0.393
ΔR2	0.13	0.082	0.274

*p < 0.05, **p < 0.01.

*β* Standardized coefficients, *NSLBP* non-specific low back pain, *LBP* low back pain, *BMI* Body Mass Index, *PCS* Pain Catastrophizing Scale, *TSK* Tampa Scale for Kinesiophobia, *PESQ* Pain Self Efficacy Questionnaire, *RMDQ* Roland Morris Disability Questionnaire, *R*^*2*^ the proportion of explained variance in dependent variable by the model, *ΔR*^*2*^ the change in R^2^ values from one model to another.

#### Acute NSLBP group

The acute NSLBP group consisted of 124 participants. Table 4 presents the results. In step 1, the demographic variables were added. The hierarchical linear regression results showed that patients' demographic factors, tested in step 1 explained 0.0% of the variance in disability. There was no significant association between all demographic factors and worse disability. In step 2, we included pain intensity as the independent variable. The results showed that the pain intensity, tested in step 2, explained an additional 7.7% of the variance in disability. There was a significant association between pain intensity and worse disability (β = 0.282; p < 0.01). In step 3, psychological factors, which were TSK, PCS and PESQ, were included as the independent variables. The results of hierarchical linear regression showed that psychological factors, tested in step 3, explained an additional 20.7% of the variance in disability. Finally, those significant relations to disability were PSEQ (β = − 0.361; p < 0.01), LBP (β = 0.280; p < 0.01) and PCS (β = 0.209; p < 0.05). This final model explained 28.4% of the variance in disability of participants.

#### Non-acute NSLBP group

The non-acute NSLBP group consisted of 111 participants. Table 5 presents the results. In step 1, the demographic variables were added. The results showed that patients’ demographic factors, tested in step 1, explained 1.3% of the variance in disability. All demographic factors were not statistically significant. In step 2, pain intensity was included as a variable. The results of hierarchical linear regression showed that the pain intensity, tested in step 2, explained an additional 8.2% of the variance in disability. There was a significant association between pain intensity and worse disability (β = 0.288; p < 0.01). In step 3, psychological factors, which are TSK, PCS and PSEQ, were included. The results of hierarchical linear regression showed that psychological factors, tested in step 3, explained an additional 27.4% of the variance in disability. Ultimately, PSEQ was significantly related to disability (β = − 0.416; p < 0.01). This final model explained 39.3% of the variance in disability of participants.

## Discussion

We examined factors associated with disability by hierarchical multiple regression analysis of the acute and non-acute NSLBP. Overall, with variables in demographic characteristics, pain intensity and psychosocial factors, the explanatory power increased. Pain self-efficacy had a significant effect in the acute and non-acute groups. From the results, the pain self-efficacy might be the critical factor associating with disability in patients with NSLBP.

For the acute NSLBP group, pain self-efficacy had the most significant association with disability. Several other studies showed the relationship between self-efficacy and disability. A study on spine disorders, including NSLBP patients, found that low self-efficacy significantly influenced disability of LBP and may also affect postoperative outcomes^26^. In a study on LBP subjects in Italy, more than half of the patients had low self-efficacy, and the group had a high level of disability^27^. It has been reported that pain self-efficacy is a more critical factor for disability than fear of movement^28^. Additionally, among fear of movement, disability and pain intensity, pain self-efficacy serves as a mediator^29^. Self-efficacy was also reported to affect functional aspects such as postural stability and range of motion at the spine; the higher the self-efficacy, the greater the stability and range of motion^10^. Based on the results of this study, it is expected that pain self-efficacy is strong association with disability. Furthermore, the significant association between self-efficacy and disability of the acute phase is one of the new findings as most of the previous studies have focused on non-acute NSLBP. Indeed, pain self-efficacy has been reported to be more critical in the recovery of LBP than other psychosocial factors, but these participants were only for the non-acute NSLBP^30,31^. The important aspect of managing NSLBP is to take appropriate action in the acute phase and prevent it from becoming chronic. However, low pain self-efficacy may lead to a vicious cycle, as typified by the fear-avoidance model, leading to chronicity. The fear-avoidance model is a model that describes how persistent musculoskeletal pain is caused and maintained as a result of fear-based attentional processes and avoidance behaviours^32^. Thus, pain self-efficacy may be a key aspect in implementing the management of NSLBP for preventing chronicity. However, one reason for the solid association between pain self-efficacy and disability may be that the PSEQ questions overlap with some disability items. For example, considering "I stay in bed most of the time because of my back" for RDQ and "I can gradually become more active, despite the pain" for PESQ, the difference between these two scales is just their response directions. Therefore, it is important to remember that it should be considered if there is a significant association.

In addition, pain intensity was indicated to be another related factor of disability for the acute phase. From previous studies, severe pain intensity is a factor in the transition from the acute to the chronic phase^4^, so improving pain intensity in the acute phase might be another key to managing chronicity.

Pain self-efficacy was also associated with disability of the non-acute phase. Previous studies have reported that many psychological factors can influence non-acute NSLBP. Ogunlana et al. showed that those with higher PCS values have a more severe disability^33^. Wertli et al. showed that catastrophising was a prognostic factor for LBP^11^. However, it has also been shown that the mechanism is still unclear. Besides catastrophic thoughts of pain, Leeuw et al. reported an association between pain-related fear and functional impairment for non-acute NSLBP patients^34^. Wertli et al. found an association between fear-avoidance beliefs and disability developed within 6 months, which is one crucial factor for chronicity^35^. These factors are also predictors of chronicity of NSLBP^36,37^. Based on the results of previous studies, it is highly likely that many psychosocial factors were related to disability for non-acute NSLBP. The results of this study suggest that pain self-efficacy may be a more relevant factor with disability.

### Limitations

One limitation of our study was the lack of clarity as to whether the participants of the acute group were truly acute in the first episode or just a recent exacerbation of a chronic problem. The duration of the disease was confirmed by interviewing the participants in this study. We did not include a question on whether it was the first time or a recurrence. Therefore, this may have overlooked the possibility that the acute group included patients with recurrence NSLBP. Second, the recruitment of participants may have been somewhat biassed, with only two facilities. If we could have recruited participants from more than two facilities, the data would have been more reliable. However, since there was no difference in the participants' characteristics between the two facilities, we believe that the sample is reliable. Third, the biopsychosocial variables were limited. Although this study selected factors that influence disability for NSLBP based on previous studies, the data might have been more attractive if other psychosocial factors such as depression and anxiety had been included. Finally, it should be noted that some of the PSEQ items and items of RDQ are overlapped.

### Clinical implication and further research

Despite the not large sample size and the cross-sectional study design with no intervention, this study revealed that pain self-efficacy is one of the most critical factors associating with disability of NSLBP. It may be helpful to examine a larger sample size and investigate the association between other psychological or social factors and disability in the future.

## Conclusion

The results suggest that the factors associating with disability differed depending on the duration of the disease, and pain self-efficacy was significantly associated with disability of NSLBP. In the future, it may be possible to investigate the association between disability and more multifaceted factors, including other psychosocial factors, to clarify the disability-related aspects of NSLBP.
